# Research Progress and Screening Strategies of Natural Product-Derived Neuraminidase Inhibitors

**DOI:** 10.3390/bios16070365

**Published:** 2026-07-03

**Authors:** Jun Duan, Xinjie Guo, Pinghua Sun, Haibo Zhou, Xiangjiu He

**Affiliations:** 1 School of Pharmacy, Guangdong Pharmaceutical University, Guangzhou 510006, China; junduan0710@163.com; 2 College of Pharmacy, Jinan University, Guangzhou 510632, China

**Keywords:** natural products, neuraminidase inhibitors, active ingredient screening, biosensors, anti-influenza drugs

## Abstract

Seasonal epidemics and high variability of influenza viruses pose a severe threat to global public health security. Neuraminidase, a key functional enzyme in the life cycle of influenza viruses, represents an important target for anti-influenza drug development. Given the continuous emergence of drug-resistant strains against first-line clinical neuraminidase inhibitors (NAIs) such as oseltamivir, there is an urgent need to develop novel, broad-spectrum, and resistance-overcoming NAIs. Natural products, characterized by structural diversity and a wide range of biological activities, provide abundant resources for the discovery of new NAIs. Recent advances in computer-aided drug design, intelligent analytical platforms, and modern screening technologies have accelerated the identification of natural product-derived NAIs. In particular, biosensor-based strategies, including electrochemical, fluorescence, bioluminescence, and surface-enhanced Raman scattering biosensors, have demonstrated significant advantages in sensitivity, selectivity, rapid response, and high-throughput screening. In combination with computational methods and experimental approaches such as affinity ultrafiltration and activity-guided separation, these technologies have promoted the development of intelligent, precise, and multimodal screening platforms. Looking forward, the integration of biosensor-based high-throughput screening platforms with artificial intelligence algorithms is expected to drive the next generation of natural product screening platforms and facilitate the efficient discovery and clinical translation of novel NAIs. This paper systematically reviews the research progress of screening strategies for natural product-derived NAIs; introduces representative natural active NAIs, including phenols, terpenoids, and alkaloids; and prospects future development directions, aiming to provide a scientific reference for the efficient discovery of NAIs from natural products.

## 1. Introduction

**Figure 1:**
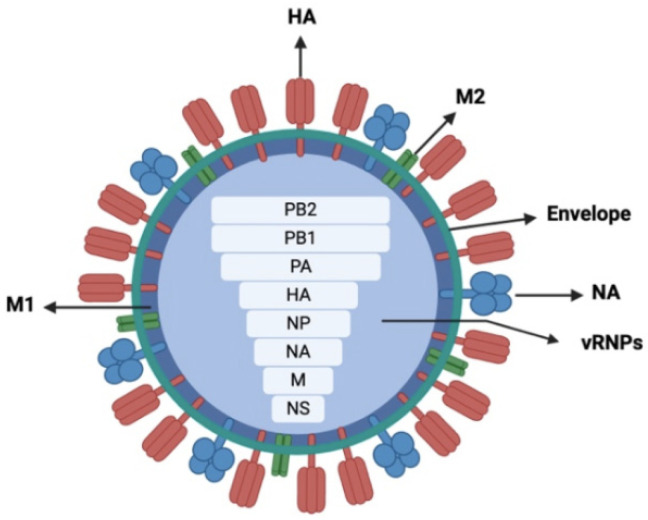
Structure of the Influenza virus. Reprinted from ref. [3].

Influenza is an acute respiratory infectious disease caused by influenza viruses, characterized by high infectivity, rapid transmission, and pronounced seasonal epidemic patterns, which seriously endangers global public health security [1]. Influenza viruses are single-stranded negative-sense RNA viruses belonging to the Orthomyxoviridae family [2]. Their structural components, from the outermost to the innermost, consist of the lipid envelope, matrix protein, and viral core [3] (Figure 1). Based on the antigenic properties of the viral nucleoprotein (NP) and matrix protein (M), influenza viruses are classified into four genera: Influenza A virus, Influenza B virus, Influenza C virus, and Influenza D virus [4].

Two major glycoproteins are embedded on the surface of influenza virus particles: hemagglutinin (HA) and neuraminidase (NA). HA mediates the initial attachment and fusion of the virus to host cell surface receptors, facilitating viral entry. In contrast, NA is a glycoside hydrolase that catalyzes the cleavage of sialic acid receptors on the host cell surface, promoting the release of newly synthesized viral particles from infected cells and preventing viral aggregation [5]. In the 1990s, based on an in-depth understanding of the structure and function of influenza virus NA, scientists developed a new class of anti-influenza drugs targeting NA: neuraminidase inhibitors (NAIs) [6,7]. However, the widespread use of NAIs has led to the emergence of various viral mutations that confer resistance to these drugs, particularly a large number of oseltamivir-resistant seasonal influenza virus strains [8]. Furthermore, the current chemical drug design based on the NA structure has reached a plateau, and the emergence of novel influenza viruses (such as avian influenza H5N1 and H7N9) has posed new challenges to the effectiveness of existing NAIs [9]. Therefore, there is an urgent need to develop novel, efficient, and drug-resistant NAIs with innovative structures and mechanisms of action [10,11].

Statistics indicate that more than 50% of clinically used drugs are derived from natural products or their derivatives [12]. Screening NAIs from natural resources offers several potential advantages, including the discovery of novel chemical scaffolds, multi-target effects, and relatively low side effects [13]. Moreover, compared with most clinically used synthetic NAIs, some natural product-derived NAIs show lower cytotoxicity, better biocompatibility, and a lower propensity to induce drug resistance, demonstrating significant advantages in the development of new anti-influenza drugs [14]. However, natural products have extremely complex chemical compositions, and the content of active ingredients is often very low. Traditional screening methods, which involve sequential extraction, separation, activity screening, and structure identification, suffer from cumbersome operations, long cycles, high costs, and the risk of losing trace active ingredients. These limitations have made it difficult to meet the demand for high-throughput screening of novel NAIs [15,16]. Therefore, facing the increasingly severe drug resistance problem of existing NAIs and the technical limitations of traditional screening methods, the development of novel, high-throughput, rapid, and effective NAIs screening methods has become an urgent priority in the field of anti-influenza drug development.

In recent years, driven by the rapid advancements in computer science, modern separation and analytical techniques, and intelligent detection platforms, a series of efficient, rapid, and highly sensitive screening technologies for NAIs have emerged. These include computer-aided virtual screening, biosensing analysis, and affinity ultrafiltration–mass spectrometry, all of which provide crucial technical support for the discovery of NAIs derived from natural products. Among these, biosensing technologies—exemplified by electrochemical, fluorescence, chemiluminescence, and surface-enhanced Raman scattering (SERS) methods—have demonstrated immense potential in activity screening, owing to their advantages of high sensitivity, high selectivity, and rapid response. This paper begins by examining the structure and function of NA, followed by a systematic review of screening strategies and novel biosensing analytical methods for natural product-derived NAIs. It summarizes the research progress regarding various classes of natural product inhibitors and offers perspectives on future trends in screening platforms—specifically their evolution toward greater intelligence, integration, and portability—with the aim of providing a valuable reference for the efficient discovery and development of natural product-derived NAIs.

## 2. Overview of NA

### 2.1. Structure and Function of NA

**Figure 2:**
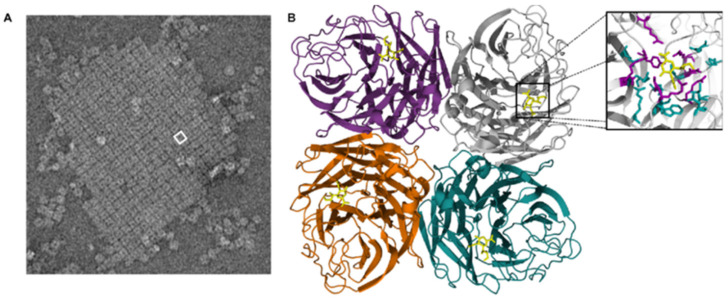
The structure of NA. Reprinted from ref. [19]. (**A**) Electron micrograph of two-dimensional crystalline arrays formed by NA heads. One square structure = one tetrameric head; the white box indicates a single tetrameric head. (**B**) Structural composition of the NA catalytic head, with an inset showing the pocket where sialic acid (yellow compound) binds.

NA is a glycoprotein present on the surface of influenza viruses. It forms a mushroom-shaped homotetrameric structure, with each subunit containing a catalytic active site at its center [17,18,19] (Figure 2). The catalytic activity of NA is co-determined by functional amino acid residues (Arg 118, Asp 151, Arg 152, Arg 224, Glu 276, Arg 292, Arg 371, Tyr 406) and structural amino acid residues (Glu 119, Arg 156, Trp 178, Ser 179, Asp 198, Ile 222, Glu 227, Glu 277, Asp 293, Glu 425). These residues are highly conserved among all NA subtypes of influenza A and B viruses, which is the key for NA to exert its catalytic function [20]. Influenza A virus NA is divided into 11 subtypes based on its antigenic properties, of which 9 subtypes have been isolated from avian hosts, while subtypes N10 and N11 are found exclusively in bats [21]. These subtypes are further classified into two groups: Group 1 includes N1, N4, N5, and N8; Group 2 includes N2, N3, N6, N7, and N9. The primary structural difference between the two groups is that Group 1 NA possesses a “150-cavity” adjacent to the active center, which is absent in Group 2 NA [22]. Due to the highly conserved structure of the active center across different NA subtypes, NA has become an ideal target for anti-influenza virus drug development [23].

**Figure 3:**
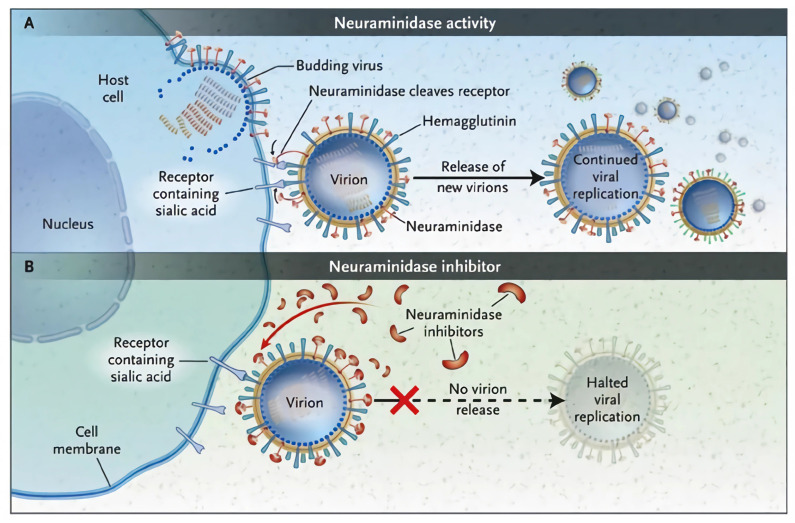
Mechanism of action of NAIs. Reprinted with permission from ref. [26]. Copyright 2005 Massachusetts Medical Society. (**A**) The role of NA in viral replication. (**B**) The effect of NAIs on viral replication.

NA plays a crucial role in the influenza virus life cycle, primarily participating in the release and transmission of viral particles. NA hydrolyzes the glycosidic bond between sialic acid on the host cell surface and viral HA, releasing newly formed viral particles from the infected cell surface, which then go on to infect new host cells [24]. Additionally, NA can hydrolyze sialic acid residues on the surface of newly synthesized viral particles, preventing viral aggregation. Studies have shown that low NA activity or NA deletion can prevent progeny viruses from being released from the host cell surface, thereby impairing their ability to infect new host cells [25]. Therefore, targeting NA with NAIs can effectively inhibit NA activity, halt viral replication [26] (Figure 3), and control the further spread of the virus in the respiratory tract.

### 2.2. Currently Marketed NAIs

**Figure 4:**
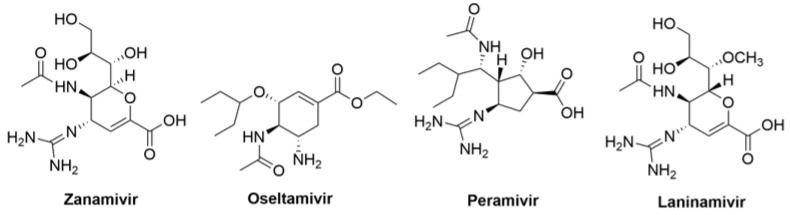
Chemical structures of NAIs as anti-Influenza virus drugs.

With advances in science and technology, the determination of the crystal structure of NA has become increasingly convenient and efficient, paving the way for the discovery and optimization of various NAIs. Currently, three anti-influenza NAIs have been approved for marketing by the U.S. Food and Drug Administration (FDA): zanamivir, oseltamivir, and peramivir [27,28]. An additional NAI, laninamivir, is approved for marketing only in Japan [29]. Their chemical structures are shown in Figure 4, all of which are derived from the structural modification of 2-deoxy-2,3-didehydro-N-acetylneuraminic acid (DANA). These drugs act by mimicking the sialic acid transition state and competitively binding to the conserved residues in the NA active center, thereby effectively inhibiting its enzymatic activity [30]. In contrast, Baloxavir marboxil (trade name Xofluza), approved by the FDA in 2018, blocks early viral genome replication at its source by inhibiting the formation of viral mRNA, representing the most groundbreaking innovation in anti-influenza drug treatment over the past two decades [31,32,33]. Although baloxavir marboxil provides a new option for clinical practice, NAIs are still the most widely used first-line treatment for influenza worldwide and have played a critical role in multiple influenza pandemics [34]. Zanamivir, the first FDA-approved NAI, was approved in 1999 for administration via inhalation. It exhibits potent inhibitory activity against both influenza A and B viruses [35]. However, inhalation administration may cause airway reactions, particularly in patients with asthma and critically ill patients [36]. Furthermore, inhalation administration results in low bioavailability and requires frequent dosing [30]. Oseltamivir was subsequently approved by the FDA and offers high oral bioavailability. Oseltamivir is a prodrug that is hydrolyzed by hepatic esterases after oral administration to its active metabolite, oseltamivir carboxylate, which exerts antiviral effects [37]. However, the widespread use of oseltamivir has led to the continuous emergence of resistant strains, significantly reducing its clinical efficacy [38]. In response to the emergence of oseltamivir-resistant strains and the clinical need for critically ill patients who cannot take oral medications, peramivir was approved by the FDA in 2014 for the treatment of acute uncomplicated influenza in children (under 2 years of age) and adults [39]. Peramivir is a long-acting intravenous NAI that retains antiviral activity against some oseltamivir-resistant influenza strains [40,41]. Laninamivir is a long-acting NAI administered via nasal inhalation. First approved for marketing in Japan in 2010 for the treatment of acute uncomplicated influenza A and B viruses, it is the only globally approved single-dose inhaled anti-influenza agent [42]. With its unique long-acting mechanism of action and excellent anti-drug resistance activity, laninamivir has become an important option for influenza treatment and post-exposure prophylaxis [43]. Nevertheless, all existing marketed NAIs face insurmountable clinical challenges: First, the problem of drug resistance is becoming increasingly severe. A single point mutation in the catalytic site can lead to a significant decrease or even complete loss of drug activity, and the prevalence of drug-resistant strains has become a major challenge in clinical anti-influenza treatment. Second, the drug structures are highly homogeneous, all designed based on the DANA, making it difficult to overcome the limitations imposed by drug-resistant mutations and resulting in insufficient broad-spectrum activity against emerging influenza subtypes. Third, the clinical application of these drugs is limited by their administration routes and adverse effects, with orally administered, inhaled, and injectable formulations all restricted in their applicable clinical scenarios. Therefore, the development of NAIs with novel structures, high efficiency, low toxicity, the ability to overcome drug resistance, and broad-spectrum activity has become an urgent need in the field of anti-influenza drugs, and natural products are the core resource to break through this bottleneck.

## 3. Screening Strategies for Natural Product-Derived NAIs

**Figure 5:**
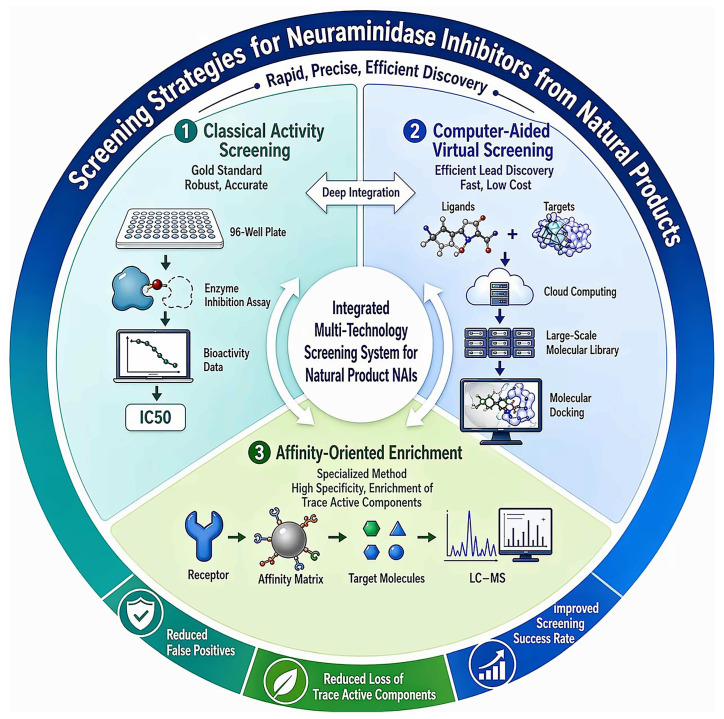
Schematic diagram of the screening strategies for NAIs from natural products.

Natural products have extremely complex chemical compositions. A single medicinal herb or extract often contains hundreds to thousands of chemical components, and the content of active ingredients is generally very low. The conventional sequential screening workflow consisting of extraction and separation, activity screening, and structural identification is plagued by inherent limitations, including low efficiency, long operation cycles, susceptibility to the loss of trace active ingredients, and a high false positive rate. After decades of technological innovation, a comprehensive screening system has been formed, with classic activity-based screening as the gold standard, novel biosensing analytical techniques as effective tools for affinity assessment, computer virtual screening as an efficient lead generation tool, and affinity-directed enrichment as a characteristic core technology. This integrated system enables the rapid, accurate, and efficient discovery of NAIs from natural products (Figure 5).

### 3.1. Activity-Based Screening Strategy

Activity-based screening is the most classic and direct screening strategy in the research and development of natural product-derived NAIs. It directly detects the inhibitory effect of natural products (crude extracts, fractions) on NA activity and influenza virus replication through in vitro or in vivo biological experiments, and obtains monomeric compounds through step-by-step separation and purification [44,45]. This strategy does not rely on target structural information or data on known active compounds, making it the core method for discovering NAIs with novel structures and mechanisms of action. With the continuous development of analytical science and detection technologies, activity-based screening strategies have gradually evolved toward greater sensitivity, rapidity, intelligence, and high-throughput capability. In particular, biosensor-based approaches have emerged as an important component of modern activity-based screening owing to their advantages in rapid signal response and sensitive detection.

#### 3.1.1. Conventional NAIs Sensing Methods

The conventional screening and sensing of NAIs has long been dominated by activity-based enzymatic assays, which remain the “gold standard” for quantitative evaluation of NAI potency. These classical methods fundamentally rely on monitoring the conversion of a substrate to a detectable product in the presence of NA and the test compound, thereby reflecting the residual enzyme activity and quantitatively determining the inhibition rate and half-maximal inhibitory concentration (IC_50_) of test compounds against NA activity [46].

The earliest NA activity assays used natural substrates, including bovine submaxillary mucin and erythrocyte membrane glycolipids. When using natural substrates to detect NA activity, the content of N-acetylneuraminic acid released after enzymatic hydrolysis is usually measured by colorimetric or fluorescence quantification [47,48,49]. However, these methods suffer from low detection sensitivity and complex operations, which cannot meet the demand for large-scale screening.

**Figure 6:**
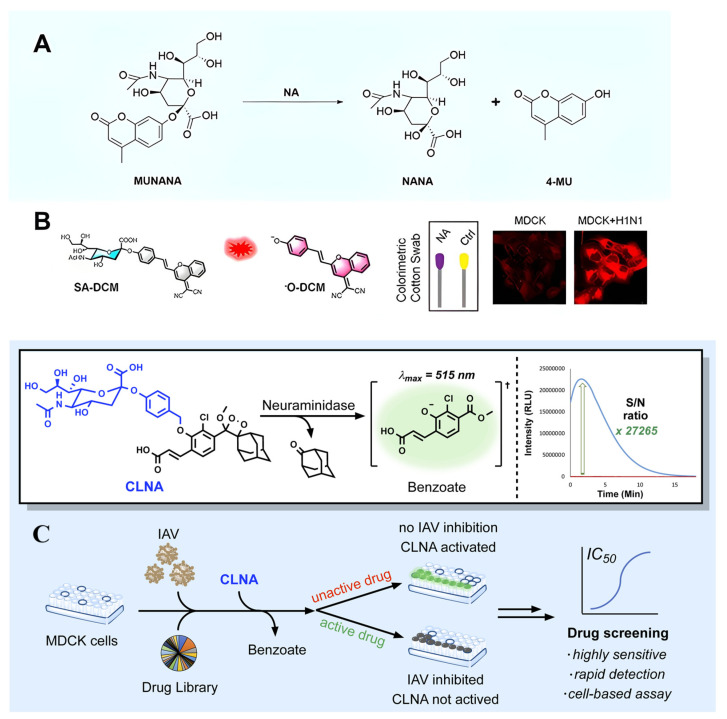
Conventional sensing methods for NA activity. (**A**) Fluorescence method, (**B**) Dualmodal ratiometric and colorimetric detection method. Reprinted with permission from ref. [51]. Copyright 2023 American Chemical Society and (**C**) Chemiluminescence method. Reprinted from ref. [52].

Subsequently, synthetic substrates were developed, which greatly improved the throughput and sensitivity of NA activity detection. Among them, the most representative is the fluorescent substrate 2′-(4-methylumbelliferyl)-α-D-N-acetylneuraminic acid (MUNANA). The 4-methylumbelliferone (4-MU) released after NA hydrolysis produces a strong fluorescent signal (Figure 6), with higher sensitivity than chromogenic substrates, a wide linear range, and good repeatability [8]. This method has been extensively validated using clinical reference inhibitors such as oseltamivir and zanamivir, yielding robust IC_50_ values and serving as the benchmark for high-throughput screening (HTS) in drug discovery pipelines. Overall, this fluorescent assay has become the most established and extensively applied method for evaluating NA activity due to its high sensitivity, operational simplicity, and robust reproducibility [50].

Overall, these developments illustrate the continuous transition of NA activity analysis from conventional enzymatic assays toward more advanced optical biosensing strategies with improved sensitivity and signal readability. Meanwhile, the integration of engineered fluorescent and chemiluminescent probes into biosensor-like platforms further strengthens their applicability for accurate, high-throughput evaluation of NAIs in complex biological contexts.

#### 3.1.2. Optical Sensing Methods

In recent years, the development of near-infrared fluorescent probes and chemiluminescent probes has further improved detection sensitivity, enabling in situ NA activity detection in vivo and the screening of low-activity compounds. Zhang et al. [51] constructed a novel NA-responsive near-infrared fluorescent probe, SA-DCM, which achieves dual-modal ratiometric and colorimetric signal output by coupling a SA recognition unit with a DCM fluorophore. This probe demonstrates significantly enhanced anti-interference capabilities and selectivity, with a detection limit reaching 5.72 × 10^−3^ mU/mL, and enables accurate imaging within live cells infected with the influenza virus. In addition, chemiluminescence detection relies on specific chemiluminescent substrates that, upon enzymatic cleavage by NA, generate an excited-state intermediate through bond breakage. As this intermediate relaxes to its ground state, photons are emitted, producing a measurable chemiluminescent signal. Owing to its inherently negligible background and signal-independent excitation, chemiluminescence affords significantly higher sensitivity than fluorescence-based methods, making it particularly well-suited for detecting low-activity samples and enabling high-precision quantitative analysis. Shelef et al. [52] developed a phenoxy-dioxetane-based chemiluminescent probe CLNA, which produces a strong emission signal at 515 nm after reaction with NA, with an extremely high signal-to-noise ratio. The detection limit for influenza viruses is 1000 times lower than that of the traditional fluorescent substrate MUNANA. Compared with reported optical probes, CLNA exhibits excellent performance in both response time and sensitivity, and is currently the most sensitive NA probe known.

**Figure 7:**
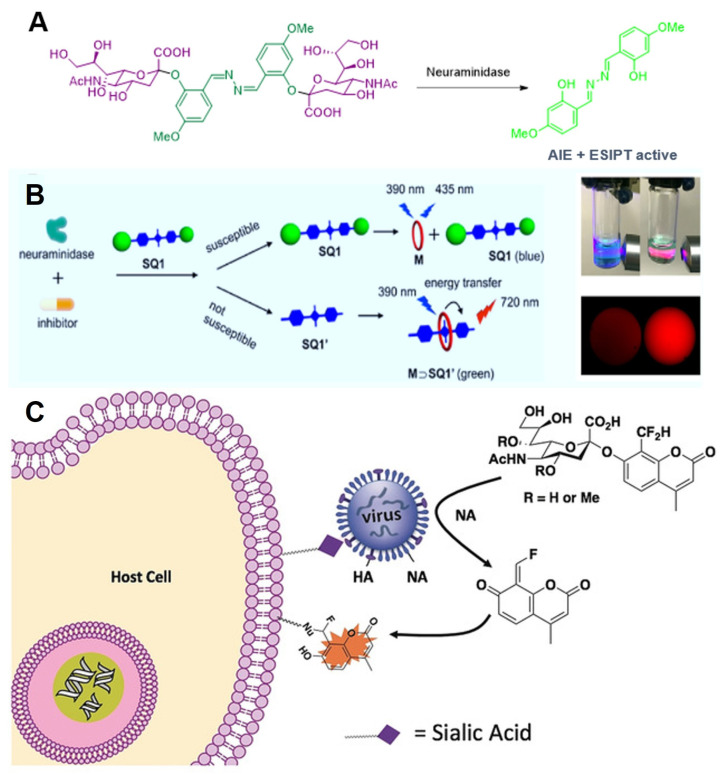
Advanced fluorescence imaging methods for NA analysis. (**A**,**B**) AIE-based assay. Reprinted from ref. [56]; (**C**) Fluorescence imaging in biological samples. Reprinted with permission from ref. [55]. Copyright 2018 John Wiley and Sons.

After the breakthrough of chemiluminescent probes in achieving ultra-low detection limits, fluorescence-based approaches continue to play a central role in NA sensing. In particular, emerging aggregation-induced emission (AIE)-based and excited-state intramolecular proton transfer (ESIPT)-based designs have expanded the functional diversity and performance ceiling of fluorescent probes (Figure 7). Chang et al. [53] developed an NA fluorescent probe SABP based on AIE and ESIPT, which uses sialic acid salicylaldazine as the NA-specific trigger group. The fluorescence signal is enhanced 30-fold in the presence of NA, with a detection limit as low as 0.024 U/mL. This probe has been successfully applied to influenza virus detection and live cell imaging, and can distinguish oseltamivir-resistant strains from wild-type viruses. The development of ultrasensitive fluorescent substrates has significantly improved detection sensitivity. Gao et al. [54] reported an ultrasensitive fluorescent reagent for NA titration, which can quantitatively detect viral NA in crude samples to the subnanomolar level. The selective design of this reagent makes it have no background response to non-viral NA, providing a powerful tool for the accurate determination of viral NA concentration. The development of fluorescence imaging technology has made it possible to visualize the spatial distribution of NA activity. Gao et al. [55] developed a proximity ligation-based fluorescence imaging reagent for NA, which labels adjacent cellular substances by releasing highly reactive fluorophores to achieve in situ imaging of enzyme activity. Moreover, this reagent can specifically detect influenza virus infection in mammalian cells, providing a new tool for the histological detection of viral infection. In addition, the introduction of supramolecular chemistry principles has brought new ideas to fluorescence detection. Liu et al. [56] reported a fluorescence NA assay based on supramolecular dye capture, which uses the host–guest interaction between squaraine derivative substrates and tetralactam macrocycles to achieve in situ capture of fluorescent products after enzymatic cleavage. This method allows for visual observation of color changes and can also be combined with affinity-capture magnetic beads to achieve diversified analysis.

In recent years, optical biosensing has further expanded toward interface-sensitive platforms based on localized electromagnetic field enhancement and refractive index modulation. Ma et al. [57] developed a method combining fluorescent paper biosensors with affinity chromatography to discover NAIs in herbal medicines, providing novel insights into the rapid screening of NAIs from natural products. Beyond conventional optical readouts such as fluorescence and chemiluminescent signals, optical biosensing has further expanded toward interface-sensitive platforms based on localized electromagnetic field enhancement and refractive index modulation. In particular, plasmonic sensing technologies, represented by SERS and surface plasmon resonance (SPR), enable highly sensitive, real-time, and label-free detection at the molecular level, providing a higher-dimensional analytical toolset for NA-related studies and inhibitor screening [58,59].

**Figure 8:**
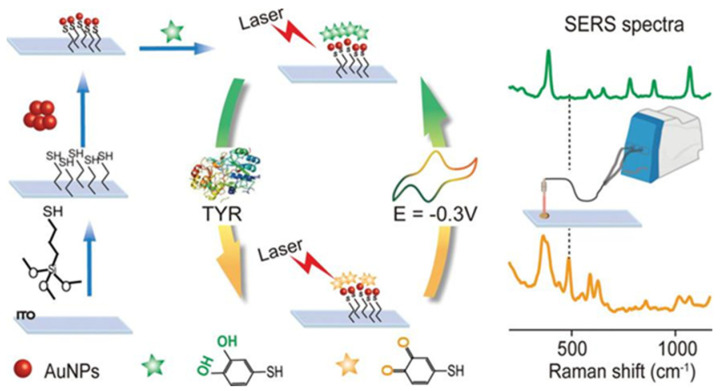
Schematic diagram of the construction of a SERS biosensor for detecting TYR activity. Reprinted with permission from ref. [58]. Copyright 2019 American Chemical Society.

As a powerful analytical technique, two main SERS-based strategies are widely employed for enzyme activity detection. The first is the direct detection strategy, also known as the label-free SERS approach. It directly monitors the changes in SERS spectra of the substrate and product during the enzymatic reaction, and leverages the intrinsic “fingerprint” recognition capability of SERS for molecular vibrational modes, thereby enabling the quantitative detection of enzyme activity and the screening of enzyme inhibitors [60,61,62]. Wang et al. [58] developed a novel and reusable label-free SERS biosensor (Figure 8). This sensor was fabricated by assembling gold nanoparticles (AuNPs) and p-thiol catechol (p-TC) on an ITO electrode, and detected tyrosinase (TYR) activity through the SERS spectral changes induced by the TYR-catalyzed conversion of p-TC to p-thiol benzoquinone (p-TB). Under optimized conditions, the developed SERS sensor exhibited a rapid response and excellent selectivity towards TYR, with a limit of detection (LOD) of 0.07 U/mL. Meanwhile, this label-free SERS approach can also be applied to NA activity detection and inhibitor screening. NA catalyzes the hydrolysis of the substrate MUNANA to produce 4-MU, which exhibits a strong Raman signal. The Raman signal intensity at its characteristic peak shows a good linear relationship with NA activity. Compared with the TYR SERS platform, only replacing the catalytic enzyme with NA is required to construct a highly sensitive SERS sensor for NA activity detection. However, how to immobilize MUNANA on the SERS substrate surface without affecting the catalytic activity is the main challenge facing the application of this method.

**Figure 9:**
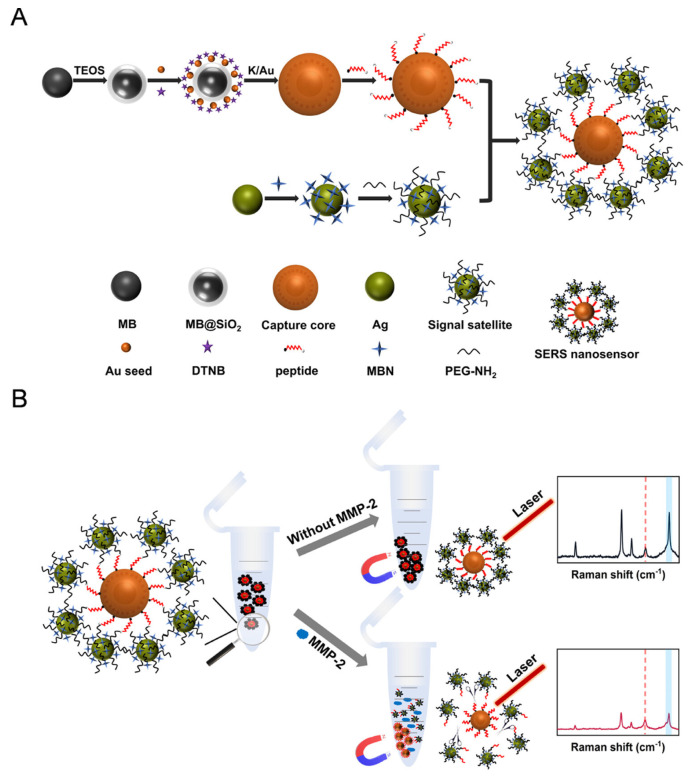
Schematic diagram of a SERS nanosensor for ratiometric detection of matrix metalloproteinase 2 (MMP-2) activity. Reprinted with permission from ref. [63]. Copyright 2024 American Chemical Society. (**A**) Fabrication of the SERS nanosensor. (**B**) Sensing mechanism of the SERS nanosensor for MMP-2 activity detection.

The second strategy is the indirect detection method, also referred to as the labeled SERS approach. This method relies on specifically designed probes conjugated with Raman reporter molecules, which are co-immobilized with the substrate on the surface of the SERS substrate. The enzymatic reaction triggers a significant change in the Raman signal intensity before and after the reaction, thus achieving the sensitive detection of enzyme activity and the screening of corresponding inhibitors [63] (Figure 9). Zhang et al. [64] established a colorimetric and SERS dual-readout assay based on the functional reporter nanoprobe 4-mercaptophenylboronic acid (4-MPBA)-modified gold–silver core–shell nanoparticles (Au@Ag NPs) for highly sensitive and selective detection of alkaline phosphatase (ALP) activity, with an LOD of 0.1 U/mL. Furthermore, this assay was also successfully applied to evaluate the inhibitory activity of ALP inhibitors. In addition, this labeled SERS approach can also be used for NA activity detection and inhibitor screening. NA catalyzes the hydrolysis of the substrate MUNANA to release sialic acid, which is then specifically captured by 4-MPBA-modified SERS probes. 4-MPBA forms boronate ester bonds with sialic acid, resulting in enhanced Raman signals. The detected Raman signal intensity of 4-MPBA shows a good linear relationship with NA activity, based on which a highly sensitive SERS sensor for NA activity detection can be constructed. However, the intensity of SERS signals is highly dependent on the uniformity of the distribution of “hot spots” on the substrate surface. Compared with ALP, NA detection requires longer incubation times, which may lead to aggregation of SERS substrates or changes in their surface properties, further reducing detection reproducibility. Developing highly uniform and stable SERS substrates is a prerequisite for achieving HST of NAIs. Taken together, benefiting from the inherent advantages of SERS biosensors in enzyme activity detection, this technology holds great promise for the highly sensitive detection of NA activity and the rapid, accurate screening of NAIs from natural products.

Beyond SERS, SPR biosensors play a pivotal role in NAI screening due to their capability for real-time, label-free monitoring of biomolecular interactions, which is highly valuable for antiviral drug discovery [65,66]. SPR detects changes in refractive index at the surface of thin metal films induced by biomolecular binding events, enabling continuous recording of interaction dynamics between immobilized targets and analytes. In NAI screening applications, NA is typically immobilized on SPR sensor chips, followed by sequential exposure to candidate compounds. The resulting sensorgrams provide quantitative kinetic information, including association and dissociation behavior, allowing comprehensive evaluation of binding affinity and interaction strength between inhibitors and NA [67]. Using this strategy, Zhang et al. [68] identified imidazo [1,2-a] pyridine derivatives as potent anti-influenza agents and further analyzed their structure–activity relationships (SARs), highlighting the utility of SPR in affinity-based antiviral screening. Furthermore, this method is also applicable to the screening of NAIs from natural products.

Apart from SPR-based systems, other biosensing technologies have also been explored for NAI screening. Nanopore biosensors, in particular, provide a powerful single-molecule approach for monitoring NA activity [69]. For instance, the cytolysin A (Cly A) protein nanopore, with an internal constriction of approximately 3.3 nm, has been used for highly sensitive detection of influenza A virus. By recording characteristic current blockade events, this system enables direct quantification of enzymatic activity through detection of products generated from NA-mediated sialic acid hydrolysis passing through the nanopore [70]. This approach can be further extended to evaluate inhibitory effects of candidate compounds, offering a versatile platform for single-molecule-level NAI screening. In addition to single-molecule biosensing strategies, the integration of biosensors with automated high-throughput platforms has further advanced NAI screening capabilities. Chen et al. [71] developed an at-line nanofractionation platform incorporating parallel oseltamivir-sensitive and oseltamivir-resistant NA bioassays, enabling rapid and simultaneous evaluation of multiple candidate compounds. This integrated system significantly improves screening throughput and efficiency, highlighting its potential for accelerating the discovery and development of anti-influenza therapeutics.

#### 3.1.3. Cell-Based Evaluation and High-Throughput Screening

Although enzyme-based inhibition assays are widely used for the primary screening of NAIs, they cannot fully reflect the antiviral efficacy of compounds in complex cellular environments. Therefore, cell-based antiviral assays are indispensable for validating the biological activity and safety of candidate compounds. The most commonly used cell-based activity assay is the cytopathic effect inhibition assay (CPE Assay). Influenza virus is inoculated into MDCK cells, and the test compound is added. The antiviral activity of the compound is judged by observing the cytopathic effect under a microscope, which is the most intuitive method for evaluating antiviral activity at the cellular level [72]. Xiao et al. [73] first screened the NA inhibitory activity of a series of 4-thiazolinone derivatives in vitro, and then evaluated the antiviral activity of highly active compounds on MDCK cells to exclude false positive results at the in vitro enzyme level. In addition, the MTT assay can be used to determine cell viability to indirectly reflect antiviral activity. Its principle is based on the fact that succinate dehydrogenase in the mitochondria of living cells can reduce MTT to insoluble blue–purple formazan crystals, and the OD value is determined by colorimetry, with higher cell viability indicating better antiviral protection effects [74]. The plaque reduction assay is a more accurate method, which precisely determines the virus titer or the antiviral activity of the drug by counting the number of plaques formed after virus infection of cells [75], but the operation is time-consuming, and the throughput is low. Compounds with excellent in vitro enzyme activity often fail to exert antiviral activity in cells due to poor cell permeability, low intracellular stability, high cytotoxicity and other problems [76,77]. Therefore, a cell-based activity assay is an indispensable key link in the screening process of NAIs, which can truly simulate the action environment of compounds in vivo, exclude false positive results at the in vitro enzyme level, and clarify the real antiviral activity and safety of compounds.

However, these conventional cell-based assays are often labor-intensive, time-consuming, and poorly compatible with the rapidly expanding scale of natural product libraries [78]. To address these limitations, HTS and high-content screening (HCS) technologies have been developed. HTS technology has high screening efficiency and can achieve full-coverage screening of large-scale natural product libraries, greatly shortening the discovery cycle of lead compounds. Based on automated liquid handling systems, high-sensitivity detection equipment, and data analysis systems, HTS technology can realize automated sample addition, incubation, and detection of large-scale samples in microplates, which greatly improves the screening efficiency [50]. Currently, a mature HTS system has been established for the activity screening of NAIs; this system is now widely employed for the preliminary screening of plant crude extracts and monomeric compounds [20,71]. Subsequently, the developed HCS technology combines automated imaging and high-throughput data analysis to quantitatively measure phenotypic changes in cells, such as morphology, survival rate, proliferation and specific biomarker expression. It can simultaneously detect multiple indicators such as antiviral activity, cytotoxicity, preliminary absorption, distribution, metabolism, and excretion (ADME) profiling of compounds at the cellular level, and obtain rich biological information at one time, realizing high-throughput screening at the cellular level [79,80].

#### 3.1.4. Electrochemical Biosensing Methods

Despite the substantial advances achieved in conventional cellular assays and HTS platforms, these methods still generally rely on labor-intensive procedures, sophisticated instrumentation, and endpoint-based analysis. Consequently, biosensor-based screening strategies have emerged as attractive alternatives for the rapid, sensitive, and real-time evaluation of NAIs [57,81]. The design principle of these biosensor-based methodologies is entirely constructed around the core biological function of NA, which specifically catalyzes the hydrolysis of α-glycosidic bonds linked to sialic acid residues. This enzymatic reaction serves as the signal transduction mediator, converting the biological catalytic event into measurable electrochemical, optical, or other types of readout signals, thereby enabling the quantitative detection of NA activity as well as the screening and evaluation of its inhibitors.

**Figure 10:**
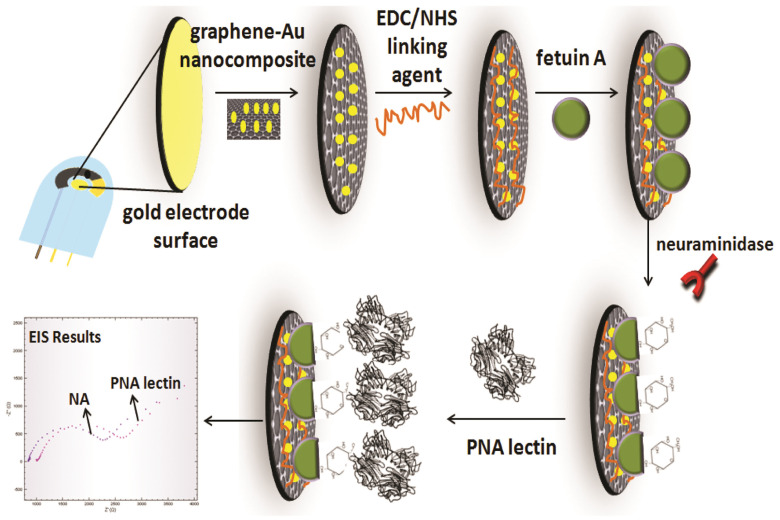
Schematic diagram of the construction of an electrochemical biosensor based on NA activity. Reprinted with permission from ref. [83]. Copyright 2018 Royal Society of Chemistry.

Electrochemical biosensors are widely used in NA activity detection and NAIs screening due to their high sensitivity, low cost and portability. In a typical electrochemical sensing strategy, NA is immobilized onto a functionalized electrode surface, and the catalytic hydrolysis of specific substrates is transduced into measurable electrochemical signals, including current, potential, or impedance variations. Upon introduction of NAIs, the enzymatic activity of NA is suppressed, resulting in decreased substrate conversion and corresponding attenuation of electrochemical responses. Such signal variations enable quantitative evaluation of inhibitor efficacy and provide a convenient platform for rapid antiviral drug screening [82,83] (Figure 10). Zhou et al. [81] developed a highly sensitive electrochemical biosensor for rapid screening of anti-influenza NAIs from Traditional Chinese Medicines (TCMs). The sensing platform was constructed by modifying a glassy carbon electrode (GCE) with a nitrogen-doped graphene/multi-walled carbon nanotube (N-Gr/MWCNTs) composite, followed by electrodeposition of polypyrrole (Ppy) to form a three-dimensional conductive network. NA was subsequently immobilized onto the carboxyl-functionalized interface via EDC/NHS-mediated covalent coupling, yielding a NA/Ppy/N-Gr/MWCNTs@GCE biosensor. Using this platform, the inhibitory activities of five classes of TCM compounds were rapidly evaluated, revealing the activity order of baicalein > baicalin > rutin ≈ matrine > ligustilide A. This study highlighted the capability of electrochemical sensing systems for rapid activity profiling and preliminary lead compound identification from complex natural product libraries.

In addition to conventional voltametric strategies, electrochemical impedance spectroscopy (EIS) has emerged as an important electrochemical biosensing modality for NA analysis [84,85]. By monitoring changes in interfacial electron-transfer resistance, EIS enables highly sensitive characterization of NA–substrate interactions without requiring additional labeling procedures. EIS is another electrochemical biosensing modality that can distinguish NAs from different pathogens and be applied to NAIs screening. Alshanski et al. [86] demonstrated that by grafting specific sialic acid derivatives onto the sensor surface and tuning its interfacial properties, EIS can be employed to detect NA activity and subsequently evaluate inhibitor efficacy. This approach is particularly crucial for identifying viral and bacterial NAs with distinct substrate binding and cleavage preferences. Furthermore, the incorporation of DNA nanomaterials has provided new opportunities for constructing advanced electrochemical biosensors. Owing to their excellent programmability, molecular recognition capability, and controllable nanoscale architectures, DNA nanostructures can effectively improve target accessibility and overcome the Debye length limitations commonly encountered in conventional electrochemical sensing systems [87]. These features contribute to enhanced signal transduction efficiency and ultrasensitive detection performance, thereby expanding the applicability of electrochemical biosensors for NAIs screening.

Compared with conventional fluorescence-based assays, electrochemical biosensors offer several notable advantages, including reduced dependence on bulky optical instrumentation, simplified operational workflows, and improved compatibility with point-of-care testing (POCT) and on-site analysis. In addition, these platforms generally maintain high sensitivity in complex biological matrices and can be readily integrated with microelectrodes, nanomaterials, and automated sensing interfaces, thereby enhancing signal amplification efficiency and analytical throughput. Nevertheless, their practical performance may still be compromised by electrode surface fouling, nonspecific adsorption, and insufficient stability of enzyme immobilization, which can affect detection reproducibility and long-term operational stability. Furthermore, challenges related to fabrication complexity, large-scale production reproducibility, and device storage stability continue to hinder their broader clinical and industrial applications. When screening NAIs from natural products, polyphenolic compounds such as flavonoids and phenolic acids, which are abundant in TCMs, exhibit significant electroactivity. They readily undergo oxidation reactions at conventional working potentials to generate background currents that severely interfere with the readout of enzyme inhibition signals based on current changes. Traditional electrochemical sensors struggle to distinguish between currents originating from changes in enzymatic reaction substrates or products and background currents produced by polyphenol oxidation [88]. To address this challenge, researchers have effectively resolved the issues of electrode fouling and polyphenol interference encountered by electrochemical biosensors in the screening of natural product-derived NAIs through the integrated application of nanomaterial interface engineering, selective permeable membrane technology, and intelligent signal analysis algorithms, providing robust technical support for the modernization of TCMs and new drug discovery [82,89,90]. Future research should further focus on the development of biomimetic recognition elements with higher specificity and integrated portable detection devices to enable on-site rapid screening [91]. To compare the detection performance of different sensing strategies for NA activity, the analytical parameters of various methods are summarized in Table 1.

Electrochemical, fluorescence, SERS, and SPR biosensors represent the four most widely utilized platforms for activity-based NAIs screening, each exhibiting distinct performance characteristics and application scenarios. Electrochemical biosensors offer advantages including low cost, rapid signal response, and strong compatibility with HTS systems; however, their performance is limited by susceptibility to interference in complex biological matrices and potential perturbation of NA activity due to redox processes at the electrode interface. Fluorescence biosensors remain the “benchmark method” for ultra-high-throughput screening (uHTS), owing to their exceptionally high sensitivity and full compatibility with automated multiwell plate readers (e.g., 1536-well formats), but their applicability is constrained by photobleaching, photostability issues, and endogenous autofluorescence in biological samples. SERS biosensors provide ultrahigh sensitivity approaching the single-molecule level and unique molecular fingerprint specificity; nevertheless, poor signal reproducibility and substrate-dependent variability remain key barriers to widespread application. SPR biosensors enable label-free and real-time monitoring of biomolecular interactions, offering detailed kinetic and mechanistic information, but are generally limited by relatively low screening throughput and pronounced nonspecific adsorption in complex matrices. In practical applications, fluorescence and electrochemical biosensors are mainly used in early-stage high-throughput primary screening due to their efficiency and cost-effectiveness. SERS-based platforms are better suited for secondary validation and mechanistic studies to eliminate false positives, while SPR biosensors are primarily applied in late-stage lead optimization to support SAR analysis through precise kinetic characterization. Taken together, the complementarity of these biosensing platforms highlights the value of integrated multimodal strategies for comprehensive NAIs screening workflows.

Overall, the activity-based screening strategy is the gold standard for the research and development of natural product-derived NAIs. Its advantage is that it does not rely on any known information and can discover lead compounds with novel structural types and action mechanisms. However, this strategy also has disadvantages such as a long screening cycle and heavy workload of separation and purification, which is usually used in combination with virtual screening technology to improve screening efficiency.

### 3.2. Target Structure-Based Virtual Screening Strategy

With the continuous expansion of natural product libraries and the increasing demand for efficient hit identification, experimental biosensing-based screening approaches are increasingly complemented by computational strategies. Target structure-based drug design (TBDD) virtual screening uses molecular docking technology to predict the binding mode and affinity of natural product molecules to the enzyme active site based on the known crystal structure of NA [92]. This method can simulate intermolecular interactions, evaluate the binding strength through a scoring function, and quickly screen potential active candidates from a large natural product library [93]. At present, a variety of natural product databases can be used for virtual screening, such as the endogenous natural product database, microbial natural product database, marine natural product database, etc. [94,95,96].

The key to the TBDD virtual screening strategy is the known crystal structure of influenza virus NA. In 1983, scientists first resolved the crystal structure of influenza virus NA, clarifying its tetrameric spatial conformation and core characteristics of the catalytic site [97]. The successful resolution of the NA crystal structure is crucial for understanding the catalytic mechanism of the enzyme, the binding mode of inhibitors, and the research and development of anti-influenza virus drugs. It reveals the conserved active site of NA, which is highly conserved among different influenza virus subtypes, making it possible to develop broad-spectrum antiviral drugs. The active site usually contains a series of key amino acid residues, such as the arginine triad (Arg 118, Arg 292, Arg 371), which bind to substrates or inhibitors through hydrogen bonds, hydrophobic interactions and electrostatic interactions. The identification of these key residues is the basis for molecular docking and pharmacophore modeling, which is helpful for the screening and design of novel inhibitors [98]. In terms of drug discovery, NA crystal structure information is the key to the development of oseltamivir, zanamivir and other drugs. However, due to the rapid mutation of the virus, the emergence of drug-resistant strains has challenged the effectiveness of existing drugs [99]. Therefore, continuous resolution of new NA crystal structures, especially from drug-resistant strains, is crucial for the development of novel inhibitors that can cope with these drug resistances. In recent years, cryo-electron microscopy (Cryo-EM) has become a key driving force in the field of anti-influenza drug development due to its significant advantage of high resolution in resolving the structure of biological macromolecules [100]. This technology overcomes the limitation of traditional X-ray crystallography on protein crystallization, especially suitable for membrane proteins and large complexes, thus accelerating the entire drug discovery process from target validation to lead compound optimization [101,102]. Cryo-EM can resolve the high-resolution structure of different NA subtypes, including the conformational characteristics of their active sites [103], which helps to understand how NAIs (such as oseltamivir and zanamivir) bind to the active site and reveal the effect of drug-resistant mutations (such as H275Y) on drug binding. Matthys et al. [104] revealed how single-domain antibodies against NA protect the body from influenza B virus infection by targeting the conserved region of NA to block the conformational change in the virus through Cryo-EM combined with single-particle analysis technology.

TBDD virtual screening methods use molecular docking technology for screening. To address the clinical therapeutic challenge posed by the oseltamivir-resistant N1-H274Y mutant strain of influenza A virus, Ha et al. [105] first employed a two-step AutoDock Vina molecular docking strategy to screen a library of 3224 flavones and flavonols from the COCONUT natural products database, and identified 163 highly active compounds with binding energies ≤−9.5 kcal/mol. Further molecular interaction analysis confirmed that flavone scaffolds bearing nitrogen-containing heterocyclic side chains and linker groups (such as ester, ether, urethane, or amide) can simultaneously anchor the sialic acid-binding pocket and the adjacent 430-cavity of NA via the Arg118-Arg292-Arg371 arginine triad, thereby achieving bifunctional anti-drug-resistant inhibition. Therefore, flavonoids are natural product scaffolds with the potential to overcome drug resistance. The advantages of molecular docking technology are fast screening speed and low cost, which can complete virtual screening of large-scale small natural product molecules in a short time, greatly narrow the scope of experimental screening and reduce research and development costs. At the same time, it can intuitively show the binding mode of compounds to NA, clarify their mechanism of action, and provide a theoretical basis for subsequent structural optimization. However, extensive validation has shown that there may be a certain deviation between docking scores and actual in vitro activity, which is prone to causing false positive results [106]. Therefore, all screening results must be verified through biological experiments.

### 3.3. Ligand-Based Virtual Screening Strategy

In contrast to TBDD virtual screening, which depends on the availability of experimentally resolved NA structures, the ligand-based drug design (LBDD) virtual screening method offers an alternative strategy that infers bioactivity from known active compounds without requiring detailed target structural information [107]. LBDD approaches utilize the structure–activity data of known ligands to construct mathematical and statistical models, from which key molecular descriptors or pharmacophore features are extracted. These models are then applied to predict the activities of unknown compounds in natural product libraries, enabling efficient identification of potential high-activity candidates [108]. Yu et al. [109] adopted a strategy combining LBDD virtual screening, molecular dynamics simulation, and bioassay to discover a novel NAIs lead compound AN-329/10738021, and obtained a high-efficiency inhibitor with an IC_50_ of 0.21 μM through structural optimization. This method does not rely on the three-dimensional crystal structure of NA and is an important supplement to TBDD virtual screening. LBDD virtual screening mainly includes similarity and substructure search, three-dimensional shape matching, pharmacophore modeling, and quantitative structure–activity relationship (QSAR). At present, the most widely used methods are pharmacophore modeling and QSAR.

#### 3.3.1. Pharmacophore Model-Based Screening

The pharmacophore model-based drug screening method was first proposed by Paul Ehrlich in 1909 [110]. The basic process is to summarize the three-dimensional structure and electronic characteristic elements (hydrogen bond donor, hydrogen bond acceptor, positive and negative charge center, aromatic ring center, hydrophobic group, hydrophilic group and geometric conformation volume collision) of molecules with specific biological activity, so as to generate the 3D conformation of ligand molecules; establish a pharmacophore training model; verify the prediction ability of the pharmacophore model using the test set; evaluate the model quality [111]; and calculate the matching degree between the screened molecules and the pharmacophore model, so as to achieve the purpose of screening. Rohini et al. [112] screened NAIs with strong inhibitory activity by a pharmacophore-based virtual strategy, and evaluated the activity of the compounds by molecular docking and molecular dynamics simulation. The advantages of pharmacophore models are fast screening speed and strong targeting, which can quickly screen compounds that meet the basic characteristics of activity from large compound libraries. Its limitation is that the accuracy of the model is highly dependent on the quality of the training set, and it is prone to false negative results for compounds with significantly different mechanisms of action and binding modes from the training set [113].

#### 3.3.2. QSAR-Based Screening

The QSAR model is a mathematical model that quantitatively reveals the relationship between the structural properties of compounds and their biological activities, including regression models and classification models. In many practical applications in recent years, using the model to predict the activity of new compounds that have yet to be experimentally determined or even synthesized is one of the most important uses of the QSAR model. It mainly collects compounds with activity data, calculates the structural characteristics of ligand molecules, selects modeling algorithms, establishes a QSAR training model, and verifies its prediction ability with a test set [114]. Lotfi et al. [115] established a reliable 3D-QSAR model of 48 compounds through computer-aided drug design, and designed novel inhibitors with higher NA inhibitory activity based on the template compounds, which were verified by molecular docking and molecular simulation, providing useful ideas for the development of novel NAIs. The most significant feature of the QSAR method is its high efficiency; especially when screening compounds in large databases, it has irreplaceable advantages. However, it also has disadvantages such as model overfitting, being prone to modeling data deviation, and activity cliffs [116].

#### 3.3.3. Artificial Intelligence Database Mining

In recent years, the rapid development of artificial intelligence (AI) technology has brought a revolutionary breakthrough to the screening of active ingredients in natural products. AI models based on machine learning and deep learning can process massive multi-dimensional drug research and development data, mine hidden laws that cannot be identified by traditional methods, and achieve efficient screening, activity prediction, mechanism of action prediction, and druggability evaluation of natural product active ingredients, which is currently a research hotspot in this field [117]. A number of large natural product databases have been built worldwide, such as the TCMD and SPECS, ChEMBL, etc., which include hundreds of thousands of natural products’ structures, activities, targets, pharmacology, toxicology and other information, providing a sufficient data basis for the training and screening of AI models [95,118]. At present, a large number of studies have successfully screened novel high-activity NAIs from natural products through AI technology, confirming the great application potential of this technology. Zhang et al. [119] constructed an NA-specific scoring function based on a Random Forest algorithm and performed efficient virtual screening of NAIs against the SPECS database. Finally, they experimentally validated and discovered two NA inhibitors with novel scaffolds, AH-034/11365875 and AH-262/08373040, from the top 100 ranked compounds, with IC_50_ values of 107.0 μM and 194.2 μM against H7N9 subtype neuraminidase, respectively. Nguyen et al. [120] trained and tested machine learning models using machine learning methods, screened the ChEMBL compound database containing nearly 2 million compounds, and found 2 compounds as potential inhibitors of influenza A and B virus NA through in vitro enzyme activity experiments. These findings indicate that AI technology has great value in accelerating the identification and screening of potential candidate drugs with anti-influenza activity. The advantages of AI database mining are that it can process massive multi-dimensional data, and the screening efficiency and accuracy far exceed traditional methods, which can realize the whole process analysis from activity prediction to druggability evaluation. Its limitations are that the performance of the model is highly dependent on the quality of training data, there is a risk of data deviation, and the interpretability of deep learning models is poor, which cannot clarify the binding mechanism between compounds and targets, and the screening results still need experimental verification [121].

Overall, both TBDD and LBDD virtual screening strategies belong to computer-aided virtual screening technology. At present, this technology has become an important way for the discovery of NAIs lead compounds, with the advantages of low cost, fast calculation speed and flexibility, and it is not affected by ligand purity, protein stability and test conditions. These in silico methods offer advantages such as low cost, rapid computation, and high flexibility, and are not constrained by experimental factors such as ligand purity, protein stability, or assay conditions. However, due to inherent limitations in scoring functions and the simplified representation of molecular recognition events, virtual screening may still generate a considerable number of false-positive hits. In this context, biosensor-based analytical platforms provide an effective experimental validation layer for rapidly assessing the binding or inhibitory potential of computationally predicted candidates, enabling early-stage discrimination of true active compounds from false positives in a rapid and sensitive manner. Therefore, the integration of virtual screening with biosensor-enabled experimental verification significantly improves the efficiency and reliability of neuraminidase inhibitor discovery pipelines.

### 3.4. Affinity Ligand Fishing-Based Screening Methods

Activity-based screening has low efficiency and heavy workload; structure- and ligand-based virtual screening have false positives; and traditional methods cannot directly identify active ingredients from crude natural product extracts. Therefore, the deep combination of multiple technologies to integrate their respective advantages is the development trend of natural product NAIs screening at present. At the same time, screening technologies for complex natural product systems have developed rapidly, realizing the direct enrichment and identification of active ingredients from crude extracts, solving the key problems of low efficiency in traditional separation and purification and easy loss of trace active ingredients. In recent years, characteristic screening technologies based on affinity ligand fishing have been developed for complex natural product systems, that is, specific enrichment of active ingredients directly from crude extracts, combined with liquid chromatography–mass spectrometry (LC-MS) to achieve structural identification [122], mainly including affinity ultrafiltration mass spectrometry, immobilized enzyme microreactor, and magnetic bead ligand fishing.

#### 3.4.1. Affinity Ultrafiltration Mass Spectrometry

**Figure 11:**
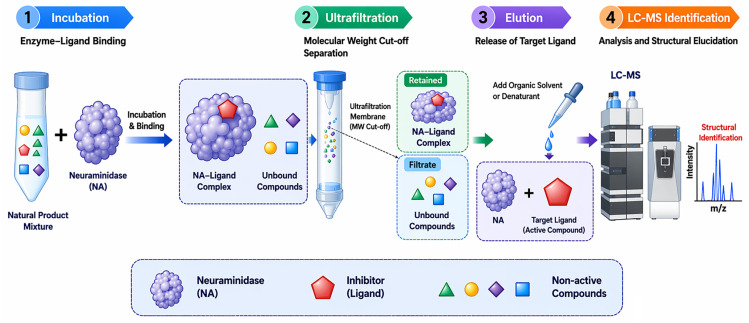
Schematic illustration of NAIs screening from natural products via AUF-MS.

Affinity ultrafiltration–mass spectrometry (AUF-MS) is a screening method that combines the advantages of affinity ultrafiltration and mass spectrometry analysis. This method incubates NA with natural product crude extracts to allow potential inhibitors to specifically bind to the enzyme. Then, an ultrafiltration membrane is used to retain macromolecules, while free small-molecule components are filtered out. Next, organic solvents or denaturants are used to dissociate the complexes and release the bound ligands. Finally, the compounds in the eluate are quickly identified by liquid chromatography–mass spectrometry [123] (Figure 11).

Tian et al. [124] used affinity ultrafiltration combined with UPLC-HR-Orbitrap-MS technology to screen 13 NA-binding small molecules reported for the first time from *Angelica pubescens*. However, traditional ultrafiltration methods have problems of non-specific binding and high false positive rates. To solve the above problems, researchers have developed various improvement strategies. Chen et al. [125] proposed an affinity interaction-guided two-dimensional separation system, which effectively reduced the interference of undissolved compounds and non-target compounds by combining affinity ultrafiltration and chromatographic separation. The introduction of molecular docking technology provides computational guidance for affinity screening. Wang et al. [92] adopted a molecular docking-guided ultrafiltration mass spectrometry combined strategy to screen neuraminidase inhibitors from *Polygonum cuspidatum*, Cortex Fraxini, and Herba Siegesbeckiae. This method first predicts the binding affinity of compounds to the target enzyme through molecular docking, and then performs ultrafiltration screening on the enriched extracts, improving screening efficiency and hit rate. In recent years, the combination of machine learning technology and affinity ultrafiltration represents the cutting-edge direction in this field. Chen et al. [126] reported a machine learning-assisted affinity ultrafiltration (ML-AAUF) strategy, which uses a gradient boosting model to predict the inhibitory activity of compounds and guide the exploration of chemical space, significantly improving the discovery efficiency of high-quality natural products. The core innovation of this method is the organic combination of computational prediction and experimental verification, realizing the transformation from “blind screening” to “rational design”.

Affinity ultrafiltration has the advantages of strong specificity, fast speed, low cost, and simple operation. Combined with LC-MS/MS, it can realize rapid separation and structural identification of active ingredients. However, this method also has some shortcomings: serious non-specific adsorption leads to false positive results; it cannot evaluate activity simultaneously; the screened components need to verify the inhibitory activity through in vitro experiments; and it has high requirements for the stability of NA, which may lead to decreased enzyme activity during incubation and ultrafiltration.

#### 3.4.2. Immobilized Enzyme Microreactor Technology

**Figure 12:**
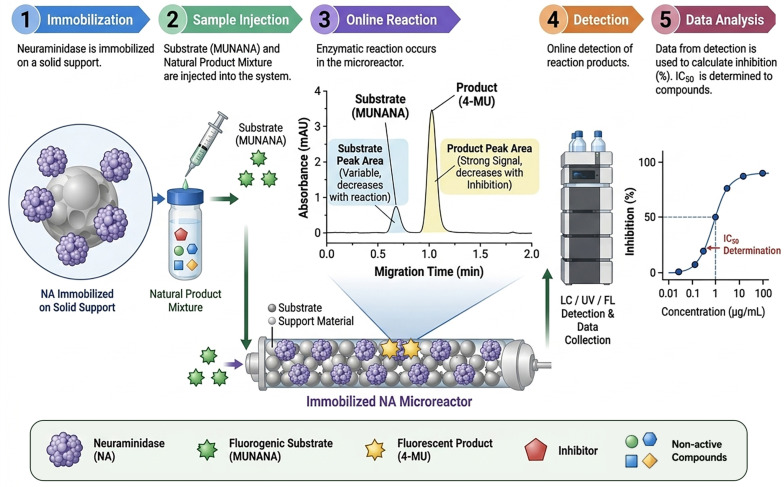
Schematic illustration of NAIs screening from natural products via IMER.

An immobilized enzyme reactor (IMER) is a reactor with catalytic activity constructed by immobilizing enzymes on carriers through physical or chemical methods. The natural product mixture and the fluorogenic substrate MUNANA are introduced into IMER, where enzymatic reactions take place within the microreactor. Affinity components (potential inhibitors) bind to NA and are thus retained on the stationary phase, achieving on-line separation from unbound components. The bound inhibitors are subsequently dissociated using organic solvents or other denaturing agents and then subjected to separation and identification via LC-MS. Meanwhile, the detector measures the peak area of the reaction product 4-MU to quantify NA activity and calculate the IC_50_ values, thereby enabling rapid and efficient screening of active compounds with anti-influenza potential. It offers advantages such as high screening efficiency, strong specificity, reusable enzymes, and low sample consumption, and has become an important technical platform for screening natural product inhibitors [127,128] (Figure 12).

Commonly used immobilization carriers include organic materials, inorganic materials, monolithic materials, and nanomaterials. Immobilization methods include physical adsorption, chemical bonding, embedding, etc. Different methods have differences in operation difficulty, enzyme activity recovery rate, stability, etc. IMER can be combined with HPLC, UPLC, CE and other chromatographic technologies to build an online screening platform for rapid screening of enzyme inhibitors from natural products [129,130,131]. Zhao et al. [128] combined CE with IMER, and prepared a NA-immobilized capillary microreactor using glutaraldehyde cross-linking technology for screening NAIs from TCMs, and found six effective NAIs from eighteen natural products. Dual-wavelength ultraviolet detection was used to eliminate the interference of screened compounds and reduce false positive results. Moreover, the immobilized capillary microreactor has good stability. After storage at 4 °C for 30 days, the activity of NA still remains 90%, and it can be used continuously for more than 200 times.

IMER has high efficiency, strong specificity, and high enzyme stability. The immobilized NA has improved tolerance to temperature and pH, can be reused, and has low sample consumption, which is suitable for screening trace natural product samples. However, its limitations include a complex preparation process and asynchronous activity verification.

#### 3.4.3. Magnetic Bead Ligand Fishing Technology

**Figure 13:**
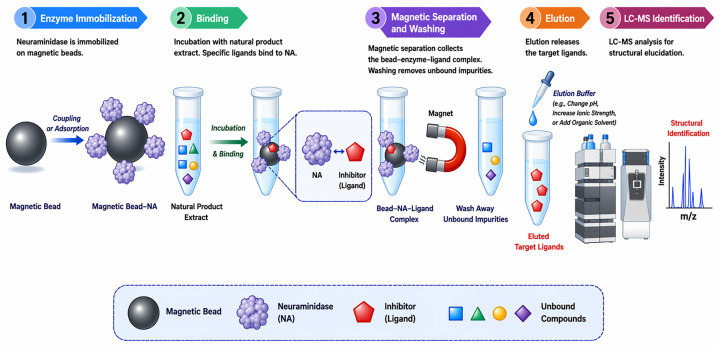
Schematic illustration of NAIs screening from natural products via magnetic bead ligand fishing.

Magnetic bead ligand fishing technology is a method of immobilizing enzymes on the surface of magnetic beads and using the superparamagnetism of magnetic beads to separate and enrich active ingredients from natural product extracts. The core principle of magnetic bead ligand fishing for active ingredients is as follows: Enzymes are immobilized on the surface of magnetic beads through covalent or non-covalent bonds to prepare enzyme-modified magnetic beads. Then, the magnetic beads are incubated with natural product extracts, and inhibitors specifically bind to the enzymes on the surface of the magnetic beads to form magnetic bead–enzyme–inhibitor complexes. Finally, an external magnetic field is used to separate the complexes from free components, elute the inhibitors with eluent, and then identify the structure by LC-MS [132] (Figure 13). Zhao et al. [46] immobilized NA on the surface of magnetic beads to prepare an enzyme microreactor, realizing the dual functions of ligand fishing and inhibitors screening. Luo et al. [133] further combined ligand fishing with high-resolution mass spectrometry to quickly identify 24 NA-binding compounds from *Duchesnea indica*.

Magnetic bead ligand fishing has the advantages of simple operation, fast speed, and strong specificity, and can quickly screen active ingredients that specifically bind to NA. However, there are certain limitations: non-specific adsorption, the immobilization process may affect the active conformation of NA and affect the binding with ligands, and some inhibitors with weak binding ability to NA are easily lost during the elution process.

### 3.5. Comparison and Future Perspectives of Different Methods

Each screening strategy has its distinct characteristics, which are systematically summarized in Table 2. Overall, conventional NAIs screening sensing methods such as the fluorogenic substrate assay are currently the most widely used and effective screening approaches. This method is mature and easy to operate, can directly reflect the actual inhibitory activity of compounds against NA, and is suitable for screening NA inhibitory activity in both natural product extracts and natural product monomer compound libraries. However, this method is susceptible to interference from background fluorescence of natural products. Biosensor technology has demonstrated significant clinical application and industrialization potential compared with traditional methods in NAIs screening, with its core advantages lying in high throughput, low sample consumption, high sensitivity, and real-time monitoring in the screening of active ingredients from natural products, and some types also possess portability or ultra-high sensitivity. Among them, electrochemical biosensors exhibit extremely strong practicality in natural product library screening due to their high sensitivity and real-time monitoring capability. Nevertheless, this method is prone to interference from polyphenolic substances in natural products, severe electrode fouling, and poor reproducibility. The virtual screening strategy eliminates the need for large-scale isolation and purification of natural products. It boasts the advantages of low cost and a broad screening scope, and can effectively narrow down the pool of candidate active compounds. Nevertheless, its screening outcomes are highly dependent on the integrity of the database and the prediction accuracy of the model, and subsequent in vitro activity assays are still required to verify the reliability of the results. Screening methods represented by affinity ligand fishing can rapidly capture active ingredients that specifically bind to NA directly from complex crude extracts of natural products. Affinity ligand fishing-based methods are most suitable for large-scale screening of complex natural product extracts and can directly achieve the separation and identification of active components. However, these methods have relatively low throughput and suffer from non-specific adsorption.

Looking ahead, we can integrate the strengths of different screening strategies to establish a multi-step combinatorial screening system. Coupled with emerging materials and analytical techniques, this system can further improve the sensitivity, specificity and efficiency of screening and facilitate the discovery of more NAIs with novel structures and excellent bioactivity from natural products. In addition, future research can pay particular attention to the interdisciplinary integration of membrane biophysics and biosensing technology to develop predictive frameworks that connect measurable physicochemical properties with the kinetics of biological processes. As demonstrated by the recent pioneering work of Wang et al. [134] in the field of membrane biophysics, based on the Collective Small Displacements model originally used to describe relaxation in glass-forming systems, they constructed a framework that can directly predict lipid transfer kinetics at membrane interfaces. Considering that NA is a typical membrane-interfacial enzyme whose function is to catalyze the hydrolysis of sialic acid residues at the interface between the viral envelope and the host cell membrane, thereby mediating the release of nascent viral particles, the physicochemical properties of the membrane environment have a significant impact on the catalytic activity of NA and the binding affinity of inhibitors. In the future, by integrating quantitative kinetic models of membrane biophysics with biosensing detection technology, it is expected to develop a new generation of predictive membrane-based screening platforms: such platforms can not only detect the inhibitory effect of inhibitors on the catalytic activity of NA, but also quantitatively predict the impact of inhibitors on NA-mediated virus-membrane separation kinetics, thereby distinguishing compounds that only have binding affinity from functional inhibitors that can truly block virus release, and providing unprecedented in-depth insights into the mechanism of action of antiviral drugs and lead compound optimization.

## 4. Natural Product-Derived NAIs

Natural products are an important treasure trove for the discovery of novel NAIs. Secondary metabolites isolated from plants, microorganisms, and marine organisms have the advantages of high structural diversity, low toxicity, multi-target effects, and low probability of drug resistance. A large number of natural products have been confirmed to have significant NA inhibitory activity, and some compounds have better activity than marketed drugs and still maintain good effects against drug-resistant strains [135]. According to chemical structure types, natural product-derived NAIs are mainly divided into four categories: polyphenols, terpenoids, alkaloids, glycosides, and compounds with other structural types.

### 4.1. Polyphenols

Polyphenolic compounds are secondary metabolites widely present in plants, containing multiple phenolic hydroxyl groups in their molecules, with structures covering flavonoids, phenolic acids, tannins, lignans, etc. They are currently the most widely studied and most reported natural products with activity [136,137]. The phenolic hydroxyl groups of polyphenolic compounds can form a large number of hydrogen bonds with the conserved amino acid residues of the NA active site, and the hydrophobic mother nucleus can bind to the hydrophobic pocket of the active site, so they have excellent NA inhibitory activity. At the same time, they have anti-inflammatory and antioxidant effects, which can alleviate lung damage caused by influenza viruses and have multi-target anti-influenza advantages [138].

**Figure 14:**
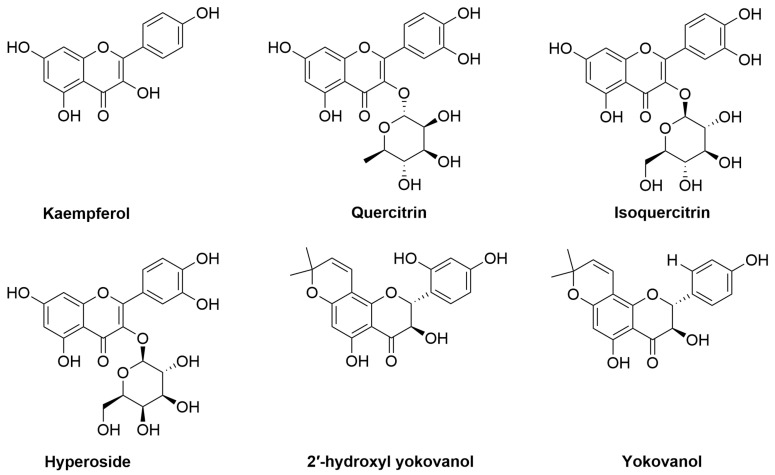
Structures of flavonoids with NA inhibitory activity from natural products.

Flavonoids are the largest class of phenolic compounds in natural products, with the basic structure of 2-phenylchromone. According to their structure, they can be divided into ten categories: aurones, biflavonoids, catechins, chalcones, flavanones, flavanols, flavans, flavones, flavonols, and isoflavones [136]. Flavonoids have a variety of biological activities, such as antioxidant, anti-inflammatory, antiviral, etc. Many of them show good NA inhibitory activity and are an important category of NAIs in natural products [139]. Kwon et al. [140] isolated six flavonoids from *Ohwia caudata* and found that two of them inhibit influenza A virus by inhibiting NA activity (Figure 14). In addition, Chen et al. [141] studied the in vitro inhibitory activity of five citrus-derived flavanones against NA, and showed NA inhibitory activity comparable to or even better than existing drugs in vitro experiments. These compounds specifically bind to the active site of NA, preventing it from cleaving sialic acid residues on the host cell surface, thereby trapping newly generated viral particles on the surface of infected cells and effectively blocking the further spread of viruses. Molecular docking and molecular dynamics simulation studies further revealed the interaction mode between these flavonoids and the NA active pocket, providing a theoretical basis for their structure–activity relationship.

### 4.2. Terpenoids

**Figure 15:**
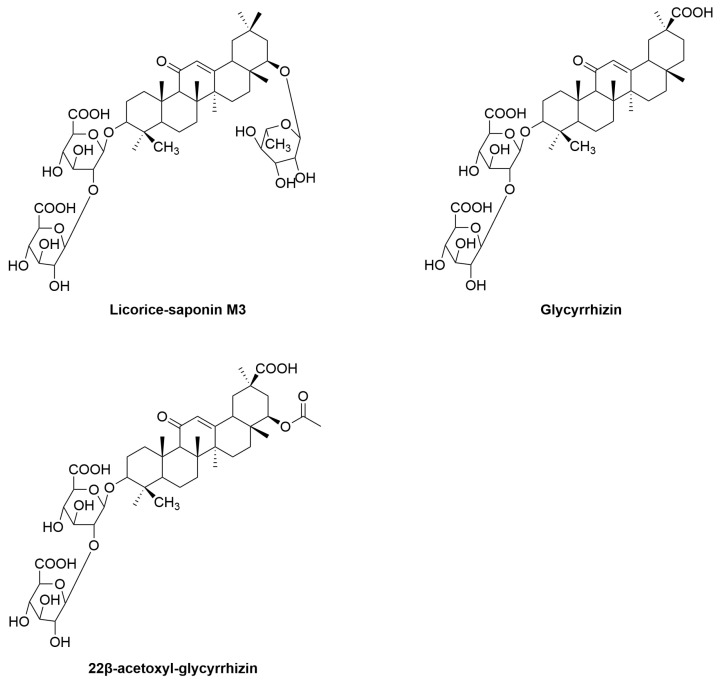
Structures of terpenoids with NA inhibitory activity from natural products.

Terpenoids are a class of natural products composed of isoprene units, widely present in plants, animals, and microorganisms. According to the number of isoprene units, they can be divided into monoterpenes, sesquiterpenes, diterpenes, triterpenes, etc. As a class of natural products with diverse structures and wide biological activities, terpenoids have shown great potential in the research and development of anti-influenza virus drugs [142]. Wei et al. [143] isolated three new oleanane-type triterpenes from the roots of *Glycyrrhiza glabra* L. In vitro NA inhibition experiments showed that five compounds had moderate inhibitory activity against NA, among which glycyrrhizin had the strongest activity, with an IC_50_ of 27.6 ± 1.1 μM (Figure 15).

### 4.3. Alkaloids

**Figure 16:**
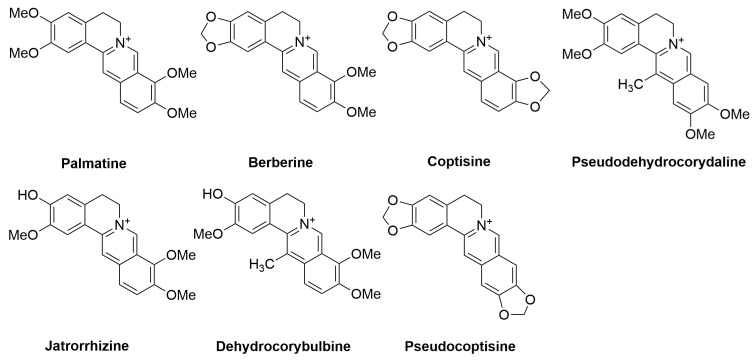
Structures of alkaloids with NA inhibitory activity from natural products.

Alkaloids are a class of basic organic compounds containing nitrogen atoms, widely present in plants, with complex structures and diverse biological activities, such as anti-inflammatory, antibacterial, antiviral, anti-tumor, etc. It has been reported that many alkaloid compounds have good NA inhibitory activity [144]. Kim et al. [145] isolated 10 alkaloid compounds from the extracts of *Corydalis turtschaninovii* rhizome, and in vitro NA inhibition experiments showed that palmatine and berberine had the strongest NA inhibitory activity. Kinetic analysis showed that both were non-competitive inhibitors. In addition, Cao et al. [146] quickly screened three alkaloid NAIs from the roots of *Toddalia asiatica* (Linn.) Lam. through the combination of ultrafiltration, chromatography and mass spectrometry (Figure 16).

### 4.4. Glycosides

**Figure 17:**
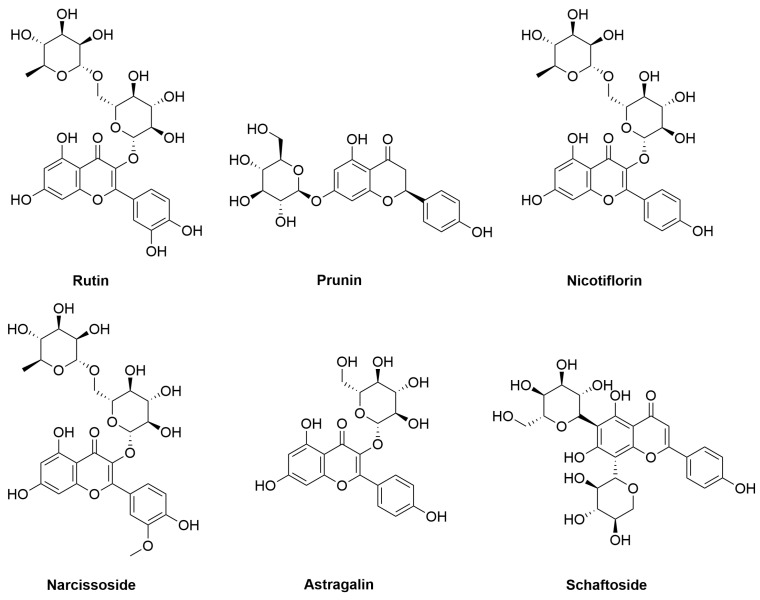
Structures of glycosides with NA inhibitory activity from natural products.

Glycosides are natural products formed by connecting sugar groups and aglycones through glycosidic bonds, widely present in plants. It should be particularly noted that glycosides are not a structural class of natural products with independent carbon skeletons, but important derivatives formed by linking one or more glycosyl groups to parent compounds via glycosidic bonds. Their parent skeletons mainly belong to structural types such as polyphenols and terpenoids discussed earlier. However, glycosylation modification, as one of the most common biotransformation methods of natural products, can significantly alter the water solubility, membrane permeability, and interaction ability with biomacromolecules of compounds. Glycosides are listed separately in this section not as an independent carbon skeleton category parallel to terpenoids and polyphenols. A large number of glycoside compounds have been confirmed to have significant NA inhibitory activity [147]. Cao et al. [148]—based on nine glycoside-containing leguminous Chinese herbal medicines, combined with network pharmacology, virtual screening, quantum chemistry and molecular dynamics simulation calculation methods—found a total of 10 compounds with NA inhibitory activity against influenza A viruses, among which 8 compounds had stronger biological activity than the positive drug oseltamivir phosphate capsules, belonging to flavonoid glycosides and dihydroflavonoid glycosides. Moreover, the study found that six of the eight natural compounds were derived from *Glycyrrhiza glabra* L. (Figure 17).

### 4.5. Others

**Figure 18:**
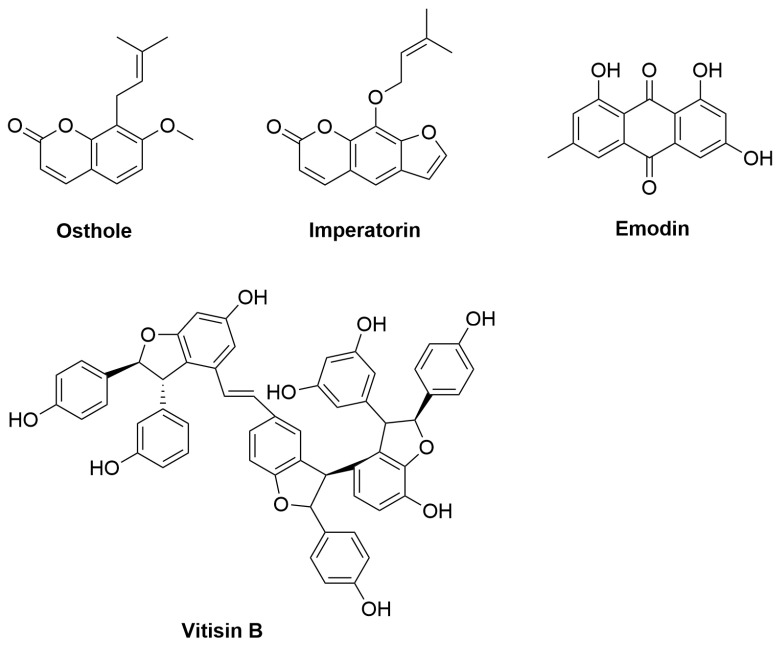
Structures of other compounds with NA inhibitory activity from natural products.

In addition to the above four categories, a variety of natural products with other structural types have been confirmed to have NA inhibitory activity, including coumarins, quinones, stilbenes, etc. Among coumarin compounds, osthole and imperatorin have good NA inhibitory activity [124]. Emodin, an anthraquinone compound, is the main active ingredient of rhubarb, with an IC_50_ value of 5.4 ± 0.1 μM against NA, showing significant NA inhibitory activity [149]. Vitisin B (VB) belongs to stilbene compounds. VB can not only inhibit the activity of influenza A virus neuraminidase and affect viral replication but also effectively inhibit oxidative stress induced by viral infection, which suggests its dual mechanism of action in influenza treatment [150] (Figure 18). This multi-target inhibition strategy is particularly important for dealing with viral drug resistance, because viruses need to overcome multiple inhibitory pathways at the same time to develop drug resistance, which is more challenging than a single-target inhibitor.

Overall, natural product-derived NAIs have core advantages such as high structural diversity, significant activity, low toxicity, multi-target effects, and low probability of drug resistance, providing abundant lead compound resources for the research and development of new anti-influenza drugs (Table 3). However, most current studies are still in the stage of in vitro activity screening and preliminary in vivo verification. The mechanism of action, pharmacokinetic properties, and druggability of most compounds are still unclear, and there is still a large gap from clinical application. Furthermore, natural product-derived NAIs still have inherent limitations, including low extraction yields, insufficient subtype selectivity, and lack of systematic SAR studies. In addition, their activity against drug-resistant mutants and synergistic effects with existing drugs remain largely unexplored, which significantly delays their clinical application process.

## 5. Conclusions

This review systematically combed the structure and biological function of NA and analyzed the clinical application dilemmas of existing marketed NAIs. It further provided a comprehensive overview of four major screening strategies for natural product-derived NAIs, including classical activity-based assays, structure-based virtual screening, ligand-based predictive modeling, and integrated multi-technology screening approaches, highlighting their respective advantages, limitations, and development trends.

Notably, biosensor-based screening strategies play an increasingly important role within this framework due to their advantages in rapid response, low sample consumption, and real-time monitoring, making them particularly suitable for crude natural product systems with complex compositions and low active ingredient abundance. Looking forward, future advancements in this field are expected to focus on multimodal integrated sensing systems to enhance matrix tolerance and analytical robustness, microfluidics-enabled ultra-high-throughput screening platforms to improve efficiency, in situ kinetic monitoring to elucidate inhibitor–target interaction mechanisms, and the integration of artificial intelligence to enable intelligent and closed-loop drug discovery.

In addition, representative classes of natural product-derived NAIs, such as polyphenols, terpenoids, alkaloids, and glycosides, were systematically categorized and discussed, underscoring the substantial potential of natural products in antiviral drug discovery. With the continuous innovation of screening technologies, the in-depth study of mechanisms of action, and the continuous advancement of druggability optimization, natural product-derived NAIs will surely play an increasingly important role in anti-influenza drug research and development, providing new solutions for responding to influenza pandemics and ensuring global public health security.

## Figures and Tables

**Table 1:** Performance comparison of sensing strategies for NA activity detection.

Modification Materials/Probes	LOD	Linear Range	Application	Sensing Principle	Refs.
SA-DCM	5.72 × 10^−3^ mU/mL	0~17.3 mU/mL	NA activity detection	Fluorescence	[51]
SABP	0.024 U/mL	0~5.0 U/mL	NA activity detection and imaging	Fluorescence	[53]
CLNA	0.13 μU/mL	1.8 × 10^−7^ U/mL~8 × 10^−5^ U/mL	NA activity detection	Chemiluminescence	[52]
Ppy/N-Gr/MWCNTs@GCE	-	-	HTS of NAIs from TCMs	Electrochemistry	[81]
AuSPE/graphene–Au hybrid nanocomposite	10^−8^ U/mL	10^−8^ U/mL~10^−1^ U/mL	NA activity detection	Electrochemistry	[83]

**Table 2:** Comparison of screening methods for natural product-derived NAIs.

Screening Methods	Advantages	Disadvantages
Activity-based screening	Direct and reliable, gold standard, can discover compounds with novel structures/mechanisms	Low efficiency, long cycle, heavy workload of separation and purification
Target structure-based virtual screening	High screening efficiency, strong targeting, can clarify the binding mode	Dependent on the crystal structure of the target, prone to false positives
Ligand structure-based virtual screening	Independent of target structure, can quickly predict activity, clarify structure–activity relationship	Dependent on known activity data, limited generalization
Affinity ligand fishing-based screening methods	Direct enrichment of active ingredients from crude extracts, no pre-separation required, high sensitivity	Prone to non-specific adsorption and protein denaturation, limited detection ability for low-affinity compounds

**Table 3:** Natural compounds with their measured NA inhibitory activity (positive control activity given in brackets).

Compound Name	Natural Resource	NA Inhibition (IC_50_ μM)	Strain	Assay Type	Refs.
Flavonoids					
2′-hydroxyl yokovanol	*Ohwia caudata*	50.8	H1N1 (zr: 10.7 μM)	FL	[141]
Yokovanol	*Ohwia caudata*	37.4	H1N1 (zr: 10.7 μM)	FL	[141]
Terpenoids					
Licorice-saponin M3	*Glycyrrhiza glabra* L.	40.0 ± 4.3	n.g (os: 1.9 ± 0.1 μM)	FL	[144]
Licorice-saponin G2	*Glycyrrhiza glabra* L.	62.5 ± 2.8	n.g (os: 1.9 ± 0.1 μM)	FL	[144]
22β-acetoxyl-glycyrrhizin	*Glycyrrhiza glabra* L.	70.8 ± 2.2	n.g (os: 1.9 ± 0.1 μM)	FL	[144]
Licorice-saponin A3	*Glycyrrhiza glabra* L.	49.7 ± 2.9	n.g (os: 1.9 ± 0.1 μM)	FL	[144]
Glycyrrhizin	*Glycyrrhiza glabra* L.	27.6 ± 1.1	n.g (os: 1.9 ± 0.1 μM)	FL	[144]
Alkaloids					
Palmatine	*Corydalis turtschaninovii*	29.8 ± 2.1	H5N1 (os: 88.2 ± 2.9 nM)	FL	[146]
Berberine	*Corydalis turtschaninovii*	32.2 ± 0.5	H5N1 (os: 88.2 ± 2.9 nM)	FL	[146]
Coptisine	*Corydalis turtschaninovii*	26.4 ± 0.1	H5N1 (os: 88.2 ± 2.9 nM)	FL	[146]
Pseudodehydrocorydaline	*Corydalis turtschaninovii*	83.6 ± 4.5	H5N1 (os: 88.2 ± 2.9 nM)	FL	[146]
Jatrorrhizine	*Corydalis turtschaninovii*	76.3 ± 2.1	H5N1 (os: 88.2 ± 2.9 nM)	FL	[146]
Dehydrocorybulbine	*Corydalis turtschaninovii*	100.2 ± 0.9	H5N1 (os: 88.2 ± 2.9 nM)	FL	[146]
Pseudocoptisine	*Corydalis turtschaninovii*	167.0 ± 1.3	H5N1 (os: 88.2 ± 2.9 nM)	FL	[146]
Haplopine	*Toddalia asiatica* (Linn.) Lam.	50.2 ± 2.6	n.g (ca: 67.8 ± 2.1 μM)	FL	[147]
Skimmianine	*Toddalia asiatica* (Linn.) Lam.	16.2 ± 0.7	n.g (ca: 67.8 ± 2.1 μM)	FL	[147]
5-methoxydictamnine	*Toddalia asiatica* (Linn.) Lam.	44.5 ± 2.7	n.g (ca: 67.8 ± 2.1 μM)	FL	[147]
Glycosides					
Rutin	*Glycyrrhiza glabra* L.	0.094	n.g (os: 127.057 μM)	FL	[149]
Prunin	*Glycyrrhiza glabra* L.	7.019	n.g (os: 127.057 μM)	FL	[149]
Nicotiflorin	*Glycyrrhiza glabra* L.	24.322	n.g (os: 127.057 μM)	FL	[149]
Narcissoside	*Glycyrrhiza glabra* L.	59.042	n.g (os: 127.057 μM)	FL	[149]
Astragalin	*Glycyrrhiza glabra* L.	64.938	n.g (os: 127.057 μM)	FL	[149]
Schaftoside	*Glycyrrhiza glabra* L.	97.275	n.g (os: 127.057 μM)	FL	[149]

(n.g = not given. zr = zanamivir. os = oseltamivir. ca = chlorogenic acid. FL = fluorescent assay).

## Data Availability

No new data were created or analyzed in this study. Data sharing is not applicable to this article.

## Abbreviations

The following abbreviations are used in this manuscript:

NAIs	neuraminidase inhibitors
HA	hemagglutinin
NA	neuraminidase
FDA	Food and Drug Administration
DANA	2-deoxy-2,3-didehydro-n-acetylneuraminic acid
IC_50_	half-maximal inhibitory concentration
MUNANA	2′-(4-methylumbelliferyl)-α-d-n-acetylneuraminic acid
4-MU	4-methylumbelliferone
AIE	aggregation-induced emission
ESIPT	excited-state intramolecular proton transfer
HTS	high-throughput screening
HCS	high-content screening
TCMs	traditional Chinese medicines
SERS	surface-enhanced Raman spectroscopy
AuNPs	gold nanoparticles
p-TC	p-mercapto catechol
ITO	indium tin oxide
TYR	tyrosinase
LOD	limit of detection
4-MPBA	4-mercaptophenylboronic acid
ALP	alkaline phosphatase
TBDD	target structure-based drug design
Cryo-EM	cryo-electron microscopy
EIS	electrochemical impedance spectroscopy
SPR	surface plasmon resonance
LBDD	ligand-based drug design
QSAR	quantitative structure–activity relationship
AI	artificial intelligence
LC-MS	liquid chromatography–mass spectrometry
AUF-MS	affinity ultrafiltration–mass spectrometry
ML-AAUF	machine learning-assisted affinity ultrafiltration
IMER	immobilized enzyme reactor
